# MELANOMAS, SARCOMAS, AND RENAL METASTASES IN THE LIVER: HOW TO TREAT?

**DOI:** 10.1590/0102-6720202400072e1866

**Published:** 2025-01-27

**Authors:** Angelica Maria LUCCHESE, Antonio Nocchi KALIL, Alessandro L. DINIZ, Karl J. OLDHAFER, Timothy M. PAWLIK, René ADAM, Olivier SOUBRANE, Maria Ignez BRAGHIROLI, Ricardo Lemos COTTA-PEREIRA, International Hepato-Pancreato-Biliary Association

**Affiliations:** 1 Irmandade Santa Casa de Misericórdia de Porto Alegre, Department of Hepato-Biliary-Pancreatic Surgery and Liver Transplantation – Porto Alegre (RS), Brazil.; 2 A. C. Camargo Cancer Center, Department of Abdominal Surgery – São Paulo (SP), Brazil.; 3 Asklepios Hospital Barmbek, Department of General and Abdominal Surgery, – Hamburg, Germany.; 4 Ohio State University, Wexner Medical Center, Department of Surgery – Columbus (OH), USA.; 5 University Paris-Saclay, AP-HP Paul Brousse Hospital, Hepato Biliary Surgery, Cancer and Transplantation Unit – Villejuif, France.; 6 Universite Paris Descartes, Institute Mutualiste Montsouris, Oncologic and Metabolic Surgery, Department of Digestive – Paris, France.; 7 Universidade de São Paulo, Institute of Cancer of São Paulo – São Paulo (SP), Brazil.; 8 D’Or Institute for Research and Education, Digestive Surgery Residency Program – Rio de Janeiro (RJ), Brazil.

**Keywords:** Neoplasm Metastasis, Liver, Melanoma, Sarcoma, Kidney, Chemotherapy, Immunotherapy, Hepatectomy, Metástase Neoplásica, Figado, Melanoma, Sarcoma, Renal, Quimioterapia, Imunoterapia, Hepatectomia

## Abstract

Liver metastases from melanomas, sarcomas, and renal tumors are less frequent. Treatment and prognosis will depend on whether they are isolated or multiple, size and location, the presence or absence of extrahepatic neoplastic disease, age, stage of the initial disease, initial treatments instituted, time of evolution, and clinical condition of the patient. Recently, a high number of oncological therapies including monotherapy or in combination, neoadjuvants or adjuvants, and immuno-oncological treatments have been developed and tested, increasing disease-free time and survival.

## RESUMO

Metástases hepáticas de melanomas, sarcomas e tumores renais são menos frequentes. O tratamento e o prognóstico dependerão se são isoladas ou múltiplas, tamanho e localização e presença ou não de doença neoplásica extra-hepática, idade, estágio da doença inicial, tratamentos iniciais instituídos, tempo de evolução e condição clínica do paciente. Recentemente, um número elevado de terapias oncológicas está disponível, monoterapia ou em combinação, neoadjuvantes ou adjuvantes, e tratamentos imuno-oncológicos foram desenvolvidos e testados, aumentando o tempo livre de doença e a sobrevida.

## INTRODUCTION

Liver metastases from melanomas, sarcomas, renal, neuroendocrine, and stromal tumors, and other tumors are less frequent compared to the incidence of liver metastases from colorectal tumors. They may be isolated or multiple. Surgical treatment, depending on the size and location and the presence or absence of extrahepatic neoplastic disease, includes hepatectomy, metastasectomy, or liver transplantation^2,14,15^. In addition to surgical treatment, in recent years, a high number of oncological therapies including monotherapy or in combination, neoadjuvants or adjuvants, and immuno-oncological treatments have been developed and tested. The prognosis and control of the disease will depend on several factors, such as age, stage of the initial disease, initial treatments instituted, time of evolution, and clinical condition of the patient^2,8,10,16^.

### Liver metastases from melanomas

Regardless of the primary tumor type, liver metastases from melanomas have a very poor prognosis, with a median survival of approximately 4 months and a 5-year survival of <5%^3^.

In a review published in 2022, Yeo et al. included studies only if they compared outcomes between surgical and non-surgical treatment for patients with liver metastases from resectable melanoma. Notably, 55.9% were cutaneous, while 37.6% were uveal. Meta-analyses showed that survival outcomes were in favor of patients undergoing surgical treatment as compared to non-surgical treatment: 1-year overall survival (OS) (hazard ratio [HR]=0.29, 95% confidence interval [CI]=0.19–0.44, p<0.00001) and 5-year OS (HR 0.07, 95%CI 0.02–0.22, p<0.00001). They concluded that surgical treatment of melanoma liver metastases could offer better OS outcomes compared with non-surgical treatment^16^.

Prolonged OS times were also reported in several non-comparative studies of hepatic resection in patients with ocular or cutaneous melanoma (range, 19-39 months). A consistent observation in these studies was that R0 resection was associated with a longer OS than R1 or R2 surgery. Other significant positive prognostic indicators included low numbers of lesions, long disease-free intervals, and limited disease distribution (i.e., unilobar)^1^.

While there is much uncertainty as to what constitutes resectable disease, characteristics such as solitary tumors and longer disease-free interval of the primary tumor to liver metastases seem to favor better prognosis and should be taken into account when determining resectability. Moreover, other conventionally negative prognostic factors such as reduced tumor size or proportion of extrahepatic disease were not found to be poorer in the non-surgical group, and the evidence for this remains unclear^16^.

Cutaneous and ocular melanomas have distinctly different clinical courses. Because there are no lymphatics in the uveal tract, ocular melanoma spreads hematogenously. Among patients with ocular melanoma who develop metastases, the liver is the predominant metastatic site (89% of cases). In contrast, metastases from cutaneous melanoma tend to spread to the lungs, lymph nodes, and soft tissue, with fewer patients (10-20%) developing liver and bowel metastases^1^.

Immuno-oncological treatments, either as monotherapy or in combination, effective in metastatic cutaneous melanomas, have not been proven to prolong survival in randomized trials or meta-analyses of patients with metastatic uveal melanoma^12^.

Although the possibilities of systemic treatment are different according to the site of the origin of the melanoma, regarding surgical treatment, the indications are similar. Studies comparing post-resection survival have shown similar results. In a study from the Melanoma Institute Australia research database, the 5-year survival rates for cutaneous and ocular primary melanomas were 44 and 39%, respectively. Hepatic resection for metastatic melanoma is associated with improved survival in selected patients with both primary ocular and cutaneous melanomas. Surgical treatment of hepatic melanoma metastases should be considered when complete resection is feasible in both^13^.

In a study by Ryu et al., the most common synchronous extrahepatic site was the lung, followed by the gastrointestinal tract, spine, lymph node, and subcutaneous tissue. Although patients with isolated hepatic metastases had a greater median survival of 44 months, compared with 12 months for patients who had extrahepatic metastases, the difference failed to reach statistical significance (p=0.20)^13^.

In a recent review conducted by Yeo et al., nine studies reported the number of patients with extrahepatic metastases during treatment. The proportion of extrahepatic metastases during treatment was greater in the surgical group (24.3%) compared to that in the non-surgical group (13.7%). They concluded that conventionally described predictors for liver metastases such as extrahepatic disease were not found to play a significant role in the prognostication of melanoma liver metastases. Thus, the evidence for tumor biology and outcomes in melanoma liver metastases still remain unclear and hence require further attention^16^.

Especially in melanoma patients, immunological treatment modalities have been studied intensively, with promising results. Recent reports showed an improved survival in patients treated with ipilimumab or activated, mutated BRAF inhibitors (including vemurafenib, dabrafenib, and encorafenib)^3^.

While there is some evidence supporting the feasibility of neoadjuvant therapy in prolonging recurrence-free survival, there remains a lack of studies comparing adjuvant and neoadjuvant therapies, and evidence for establishing a neoadjuvant regimen remains inconclusive, according to Yeo et al.^16^.

Gorry et al. conducted a Cochrane review published in 2023. This review systematically appraises the literature investigating the use of neoadjuvant treatment for stage III and IV cutaneous melanomas. They concluded that they are uncertain whether neoadjuvant treatment increases OS or time for recurrence, compared with no neoadjuvant treatment, and it may be associated with a slightly higher rate of adverse effects. There is insufficient evidence to support the use of neoadjuvant treatment, in clinical practice^7^.

## CONCLUSIONS AND AUTHOR’S COMMENT

Timothy M. Pawlik: It is pretty rare. I think there’s probably a difference between ocular and cutaneous melanoma. I mean, we think that ocular melanoma has a particular tropism for the liver and, and that is kind of a preferred metastatic site for ocular melanoma. We published a paper like 15 years ago that showed that outcomes after resection for ocular melanoma were slightly better than for cutaneous melanoma. I think right now patients are treated with systemic targeted therapy or if patients have a large burden of disease in their liver they will get isolated perfusion therapy. But I think resection is exceedingly rare. It’s challenging, but we do use an isolated hepatic perfusion approach for patients who have uveal melanoma in the liver.”

Rene Adam: I think liver metastasis from melanoma is not really a good surgical disease, the results are very disappointing. When we see the results, it’s an older study that we did 15 years ago, but it’s still true, I would say the hope of long term survival in either cutaneous or coroid melanoma is around 20%. This is a very good example of the multidisciplinary team, the decision should not be taken by the surgeon, the decision should be taken by a multidisciplinary team.

Maria Ignez Braghiroli: Cutaneous melanoma is totally different from uveal melanoma where immunotherapy does not play a role, I mean, an important role in the treatment, it doesn’t have the same activity in uveal melanoma compared to cutaneous melanoma. So, we have seen a complete change in how to manage this disease over the past 10 years since some patients do respond and some of them do respond pretty well to immunotherapy. But I would think of a patient who has a very good response to immunotherapy has a sustained response to immunotherapy and maybe hasn’t relapse in the liver or somewhere else. I would think of local treatment for this patient who has a specific relapse to treatment and then maybe it could be in the liver. But apart from that, I think systemic treatment is specifically for melanoma is the mainstay.

Olivier Soubrane: Probably are very unusual and rare tumor, very rare. Indications are for very selected patients.

Karl J. Oldhafer: The problem is that we often find multiple lesions, one millimeter, two millimeters milder model of metastatic in the liver. We thought before operations that it is maybe a single single tumor, but we find more than than one. So the question is how can we trust in MRI (magnetic resonance image) or is it necessary to perform a laparoscopy before to figure out if we have a different stadium of the tumor? We cannot cure the patient, we are able to give the patient a longer survival. If you talk about ocular melanoma patients. So if we have a patient who has liver metastases due to ocular melanoma, if it’s liver only, we do first liver resection, if it’s possible, or second, we do liver perfusion with mitomycin.

### Liver metastases from renal tumors

Liver is the third most common site of metastasis of renal cell carcinoma, occurring in 19% of cases. Treatment of metastatic renal cell carcinoma (mRCC) has evolved with the development of a variety of systemic agents; however, these therapies alone rarely lead to a complete response^10^.

Lyon et al. (cited by Mikhail et al.^10^) recently examined survival outcomes among patients with mRCC treated with or without complete surgical metastasectomy (SM) during an era when targeted therapy and checkpoint inhibitors were available. They reported a greater 2-year cancer-specific survival in patients who underwent complete SM than in those who did not (84 vs*.* 54%, p<0.001). They concluded that metastasectomy may be considered for appropriately selected patients, even in the post-cytokine era. Surgical resection may be beneficial given that bulky tumors can inhibit immune responses that are vital to combating cancer^10,11^.

Selection is critical to achieving optimal outcomes, and liver metastasectomy for mRCC is one of the best examples of this surgical maxim. The great challenge is to establish which patients do well with resection. Several factors have been identified to better define surgical indications. The last three reviews on this subject, published in 2020, 2021, and 2022, by Hall et al., Martel et al., and Mikhail et al., respectively, all found the following prognostic factors: primary tumor grade, synchronicity, complete resection, extrahepatic disease, interval from nephrectomy to liver metastasectomy, multivisceral metastasis resection, and Eastern Cooperative Oncology Group (ECOG) status. A multidisciplinary approach is essential for the therapeutic decision in order to expose to risk only those patients who really seem to benefit from surgery^4,9,10^.

The treatment of mRCC has undergone an impressive transformation over the last decade with a high number of oncological therapies available. It seems that the best results are achieved by combining immunotherapy and surgery. However, these results, although encouraging, are still preliminary. In any case, it is noteworthy that the ideal sequence for systemic and surgical therapy still needs to be defined.

## CONCLUSIONS AND AUTHOR’S COMMENT

Maria Ignez Braghiroli: Kidney cancer has a very intimate relation with our immune system. It’s not uncommon to see a very indolent disease. So, I do think we consider local treatment, specially for one isolated nodule either in the liver or in the pancreas.

Rene Adam: One point that is important about kidney metastasis for the liver is the disease free interval between the primary kidney tumor and the liver tumor. The longer is the interval, the best is the prognosis. The other point is the possibility to perform a R0 resection. The key is the control of the disease by the systemic treatment.

Antonio Nocchi Kalil: We have more metastasis to the pancreas than to the liver in our experience. We do perform liver resection for renal cancer, and we see a high incidence of recurrence.

### Liver metastases from sarcomas

Sarcomas are rare mesenchymal tumors that comprise more than 70 subtypes and have a propensity for hematogenous metastases. Hepatic metastases are uncommon for soft tissue tumors of the extremities but may be observed in approximately 16% of patients with retroperitoneal sarcomas and 62% of patients with visceral sarcomas. Gastrointestinal stromal tumor (GIST) is the most common histologic subtype of sarcomas and the most common source of hepatic metastases^5,8^.

For non-GIST soft tissue sarcomas, since current chemotherapy or other treatment options do not lead to cure, resection of liver metastases should be considered and discussed with a multidisciplinary team for all patients with technically resectable metastases as a potential treatment option^8^. It is pivotal to meticulously select patients who are candidates for potentially curative resection. Ecker et al. affirmed that to determine candidacy for hepatic resection for non-GIST sarcomas, we should follow the indications for liver metastases from other primaries tumour, analyzing the hepatic invasion of disease that can be removed, preserving an adequate hepatic reserve. Also, that extrahepatic disease is not necessarily a contraindication for surgical therapy as long as the overall treatment plan addresses all the tumor sites^5^.

For GIST, the critical question in considering metastasectomy is determining whether surgery provides any additional benefit over remaining on standard-of-care tyrosine kinase inhibitor (TKI) therapy alone. Fairweather et al. found that metastasectomy should be considered in patients with responsive or stable disease with TKIs while the disease is under control and may also be beneficial for those with unifocal progressive disease to delay switching to sunitinib^6^. After starting imatinib treatment, surgical resection is generally recommended among responders within 9 and 12 months^15^. In selected patients, resection is sometimes performed just to remove a progressing tumor (i.e., resistant clones to tyrosine kinase inhibition), especially when the patient is symptomatic, while leaving macroscopic disease that is still responsive to therapy. Regardless of the strategy chosen, tyrosine kinase inhibition is continued indefinitely for patients with metastatic GIST, irrespective of whether their disease is resected^5^.

## CONCLUSION AND AUTHOR’S COMMENT

Rene Adam: Drug treatment for GIST is very effective. And what we have learned in the past is that these patients should be treated for a prolonged time with Gleevec^®^ (imatinib mesylate). But sometime we may be faced with a clone, a tumor, which may being in progression, while all the other are well controlled by the treatment. At that time, exceptionally, it could exist an indication for a local treatment of such type of clonal. But what I would say that something which is still not totally clarified is whether there is an indication for surgery for residual tumor in the liver which is very well controlled by the Gleevec^®^. We have no clear proof that to do surgery for these residual tumor when stabilized with Gleevec^®^ is really useful or not. My trend will be to say, possibly yes, but I have no proof of that.

Olivier Soubrane: We perform upfront treatment with tyrosine kinase inhibitor. I think, but the in very few cases, there will be some room for surgical resection.

Maria Ignez Braghiroli; I think the evidence is pointing out to the longer you keep them on medication, specifically, if they have the metastatic disease, the better control will have.

Timothy M. Pawlik: Sometimes patients have great responses to Gleevec^®^. But then over time they develop like a resistant clone. And then they may have like local progression at one particular site in their liver. So, after discussing those patients in a multidisciplinary conference, if there’s a sense that they have local progression at one particular area in their liver, then I think it can be targeted for local regional therapy, whether that be resection or ablation or whatever.
